# Establishment of a nomogram model for acute chest pain triage in the chest pain center

**DOI:** 10.3389/fcvm.2023.930839

**Published:** 2023-03-21

**Authors:** Na Yan, Ling Wei, Zhiwei Li, Yu Song

**Affiliations:** ^1^ Department of Emergency, TEDA International Cardiovascular Hospital, Clinical School of Cardiovascular Disease, Tianjin Medical University, Tianjin, China; ^2^ Department of Emergency, TEDA Hospital, Tianjin, China; ^3^ Department of Pathophysiology, State Key Laboratory of Medical Molecular Biology, Institute of Basic Medical Sciences Chinese Academy of Medical Sciences, School of Basic Medicine Peking Union Medical College, Beijing, China

**Keywords:** acute chest pain triage, acute myocardial infarction, troponin, point-of-care (POC), acute coronary syndrome

## Abstract

**Background:**

Acute myocardial infarction (AMI) is the leading life-threatening disease in the emergency department (ED), so rapid chest pain triage is important. This study aimed to establish a clinical prediction model for the risk stratification of acute chest pain patients based on the Point-of-care (POC) cardiac troponin (cTn) level and other clinical variables.

**Methods:**

We conducted a *post-hoc* analysis of the database from 6,019 consecutive patients (excluding prehospital-diagnosed non-cardiac chest pain patients) attending a local chest pain center (CPC) in China between October 2016 and January 2019. The plasma concentration of cardiac troponin I (cTnI) was measured using a POC cTnI (Cardio Triage, Alere) assay. All the eligible patients were randomly divided into training and validation cohorts by a 7:3 ratio. We performed multivariable logistic regression to select variables and build a nomogram based on the significant predictive factors. We evaluated the model's generalization ability of diagnostic accuracy in the validation cohort.

**Results:**

We analyzed data from 5,397 patients that were included in this research. The median turnaround time (TAT) of POC cTnI was 16 min. The model was constructed with 6 variables: ECG ischemia, POC cTnI level, hypotension, chest pain symptom, Killip class, and sex. The area under the ROC curve (AUC) in the training and validation cohorts was 0.924 and 0.894, respectively. The diagnostic performance was superior to the GRACE score (AUC: 0.737).

**Conclusion:**

A practical predictive model was created and could be used for rapid and effective triage of acute chest pain patients in the CPC.

## Introduction

Acute myocardial infarction (AMI) is a very common cause of hospital admission in the emergency department (ED) and causes heavy healthcare burdens (1). AMI is caused by a luminal thrombus or a sudden plaque hemorrhage imposed on an atherosclerotic plaque (2). The early diagnosis of acute coronary syndrome (ACS) is important for the management of acute chest pain patients. An early revascularization procedure could reduce the mortality of AMI patients (3–5), and for non-AMI patients, an early diagnosis could reduce the potentially avoidable hospital admissions.

Point-of-care (POC) cardiac troponin (cTn) is widely used and plays a critical role in chest pain centers. Although high-sensitive cardiac troponin (hs-cTn) in the central laboratory could provide better diagnostic accuracy, it requires a longer turnaround time (TAT) of approximately 1 h in most hospitals (6, 7). The TAT includes the time of registering patient information in the hospital information system, transferring the sample to the clinical laboratory, centrifuging the samples, performing the assay, and reviewing the result. The 0/1 h algorithm with hs-cTn is recommended by The European Society of Cardiology (ESC) guidelines (8). However, hs-cTn is majorly used for rule-out AMI, not for rule-in AMI. Many factors such as renal dysfunction could cause false positive results (9), and most internists do not use cTn only as the reference biomarker (10). Serial testing has better diagnostic accuracy but requires more time and cannot meet emergency needs for acute chest pain triage. A guideline-compliant POC cTn assay should have a rapid TAT of less than 1 h (11). Previous research found that the diagnostic accuracy of POC cTn is satisfactory in aiding clinical decisions, reducing unnecessary hospital admissions (12, 13), and reducing care time in ED (14).

The POC cTnI assay is routinely used in the chest pain center (CPC), which is a regional chest pain center unit intended to get faster and standardized management of suspected ACS patients in the emergency department. Chest pain patients in the emergency department will undergo a preliminary screening according to medical history, risk factors, symptoms, and physical examination, and only approximately 5% of patients who are suspected high risk (including five fatal diseases: STEMI, NSTEMI, unstable angina pectoris, aortic dissection, and pulmonary embolism) are admitted to the CPC for further triage. Moreover, the chest pain center is not limited to China but is an important organization worldwide such as in the United States (15), Germany, and other European countries (16, 17), which are called chest pain units. POC cTnI assays are less sensitive than that of the central laboratory and may get a false negative result in the early stage of acute myocardial infarction (AMI). The diagnosis of AMI is usually based on the clinical history, electrocardiogram, and an increase in cardiac troponin concentration (8). However, this highly depends on personal experience. Therefore, using a prediction model with the combination of these factors may provide better diagnostic accuracy. Previous studies have reported that the Troponin-only Manchester Acute Coronary Syndromes (T-MACS) model could aid decisions on acute chest pain patients in the emergency department (12, 13), but it remains unknown if such a model can be generalized for other populations in chest pain centers/units.

In this study, we have established a simple risk stratification model through retrospective analysis of the clinical data of a large-scale population in CPC, which could be used for rapid and effective triage of acute chest pain patients.

## Methods

### Study population

This is a retrospective study. Between 1 October 2016 and 31 January 2019, all patients attending the CPC of TEDA International Cardiovascular Hospital were included in this study. Patients with high-risk chest pain (suspected ACS, aortic dissection, or pulmonary embolism) in the emergency department attended the CPC. Inclusion criteria were as follows: acute chest pain (onset time <24 h) patients attending the emergency department or suspected high-risk chest pain patients transferred from other hospitals in surrounding cities and regions. Exclusion criteria were as follows: Prehospital-diagnosed non-cardiac chest pain (NCCP) patients. Because of the large number of chest pain patients in our center every day (more than 100 people per day), to reduce the queuing time and to leave time for emergency care of fatal chest pain patients, prehospital triage was performed by the attending clinician in the emergency department according to symptoms and medical history, and NCCP patients didn't attend the CPC. Most patients can be diagnosed with non-cardiogenic chest pain by simple physical examination and consultation, such as herpes zoster caused by skin surface herpes. There are fixed tender points in the costal cartilage of patients with costochondritis. In patients with chest pain caused by pneumothorax, unilateral lung breathing sound is weakened or disappeared; in patients with transient pain sites, pinprick pain lasting 1–2 s at a time is usually a disorder of vegetative nerve function.

All patients were randomly divided into the training and validation by a ratio of 7:3 using random sampling. The data were exported from the database of the CPC server. This is a regional chest pain center unit in Tianjin Economic-Technological Development Area.

Electrocardiogram (ECG) was requested immediately on arrival at the CPC. Patients with suspected ACS arrived at the CPC in three ways: (a) by ambulance from any of the 72 local hospitals, (b) called for an ambulance from home, or (c) came to the hospital by themselves. The diagnosis of AMI was made by clinicians with at least one of the following: (a) Coronary arteriography (CAG) indicating a flow-limiting dysfunction such as coronary dissection, thrombotic occlusion of the epicardial artery, disruption of collateral flow, or distal embolization. (b) For patients that did not undergo CAG, checking for ischemic symptoms and typical ECG changes of STEMI or abnormal Q waves, or serial changes in high-sensitive (hs)-cTnI/T levels and echocardiography/computed tomography (CT)-based coronary angiography showing myocardial infarction (MI) (18).

### Laboratory measurement

EDTA anticoagulant whole blood samples were collected immediately on arrival at the CPC, and the POC cTnI was measured with a Triage cardiopulmonary Function Test Kit panel (Huan Zhong Biotechnology Co. LTD, HeiBei, China) on the Biosite Triage® Meter Plus System (BIOSITE Inc., San Diego, CA, United States). The test panel contains five assay items using fluorescence immunoassay, including B-type natriuretic peptide (BNP), troponin I, creatine kinase (CK), creatine kinase-MB (CK-MB), and myoglobin, and the result is available within 15 min. According to the manufacturer's instructions, the analytical characteristics of cTnI using the kit were as follows: (1) linear range: 0.05–30 ng/ml, *r*^2 ^> 0.99; (2) repeatability: coefficient of variation (CV) %<15%; (3) limit of detection (LoD) <0.05 ng/ml; (4) normal range: using 95% confidence intervals, POC cTnl reference values in the normal population were less than <0.4 ng/ml.

EDTA anticoagulant whole plasma samples (centrifugated at 3,000 rpm for 15–20 min) were used for highly sensitive troponin assay using the access hsTnI kit (catalog #B52699, Beckman Coulter, CA, United States) on the Unicel DxI 800 immunoassay analyzer (Beckman Coulter, CA, United States). The guidelines of the International Federation of Clinical Chemistry (IFCC) have recommended the analytical performance standard of high-sensitivity cardiac troponin assays (19–21). The analytical performance of access hsTnI was in accordance with the IFCC recommendations (22–24). According to the manufacturer's instructions, the Limit of blank (LoB) was 1.2 pg/ml, LoD was 2.0 pg/ml, and Limit of quantitation (LoQ) was 2.1 pg/ml. The 99-percentile upper reference limit (URL) was 17.5 ng/L with a CV of 3.7%. The sex-specific 99-percentile URL was 19.8 ng/L (male) and 11.6 ng/ml (female).

### Statistical analysis

Statistical analyses of the baseline characteristics were done using SPSS V.22.0 (Chicago, IL, United States). Categorical variables were reported as counts and percentages and were analyzed with one-way Pearson's *χ*^2^ test. Normality was determined using the Shapiro–Wilk test and all variables analyzed were not normally distributed. Descriptive statistics for continuous variables which were not normally distributed were reported as quartiles (Q1 and Q3) and compared between the training and validation set with the Mann–Whitney test. To select variables for the prediction model, logistic regression was performed, and variables that were statistically significant in univariate analysis were chosen to construct the multivariate model. The following statistical analysis and plotting were conducted with the R software (version 4.0.2). To visualize the expression and diagnostic value of cTnI, the violin plot and ROC curve were plotted with the ggplot2 package (v3.3.2) and pROC package (v1.16.2), respectively. To construct the prediction model, we used the glmnet package (v4.0.2) in the binomial model with the selected variables in the multivariate analysis. The glmnet package default uses the least absolute shrinkage and selection operator (LASSO) analysis in a penalized logistic regression model (25). We performed the prediction (high AMI risk or not) using the prediction function of the stats package (v4.1.0) to validate the model in the training and validation set. The ROCR (v1.0-11) R package was used to calculate the diagnostic performance (true positive rate or false positive rate) of the prediction model with prediction and performance function, and the ROC curve was plotted with the plot function.

## Results

**Figure 1:**
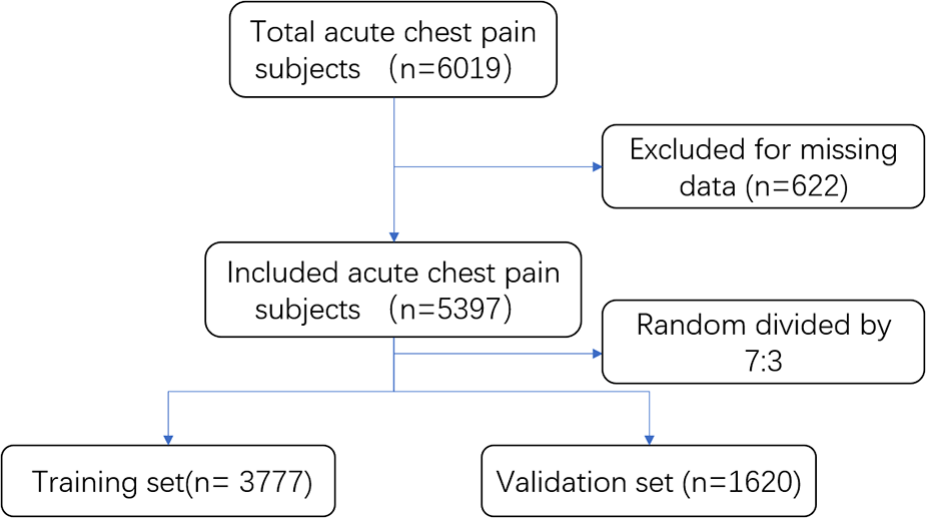
Flowchart for the selection of study participants.

We collected data from 6,019 consecutive patients in the study. After excluding patients with missing data, 5,397 patients were included in the final analysis. Included patients were randomly divided into training and validation cohorts by a ratio of 7:3 (Figure 1). The population contains 2,667 STEMI, 1,259 NSETMI, 658 UA, and 813 noncardiac chest pain (including 73 pulmonary artery embolisms and 90 aortic dissections) patients. The clinical characteristics of the patients in the two groups are shown in Table 1. The baseline data were not significantly different except for the time from symptom onset to arrival in the ED and the POC cTnI level. Considering the detection window phase of cTnI, a shorter time interval may cause inferior diagnostic performance. However, the difference is small, and the time is above 3 h with a median value of 210 min in the training set and 192 min in the validation set, so we think the effects on the model may be negligible.

**Table 1:** Clinical characteristics of all patients.

Characteristic	Total (*n* = 5,397)	Training cohort (*n* = 3,777)	Validation cohort (*n* = 1,620)	*p*-value
Age, years	62 (54, 70)	62 (54, 70)	62 (54, 71)	0.272
Sex, male, %	3,847 (71.3)	2,702 (71.5)	1,145 (70.7)	0.523
Symptom, %	/	/	/	0.588
Relieved or nontypical chest pain	1,203 (22.3)	828 (21.9)	375 (23.1)	/
Intermittent chest pain	2,345 (43.5)	1,653 (43.8)	692 (42.7)	/
Persist chest pain	1,849 (34.3)	1,296 (34.3)	553 (34.1)	/
Transferred on ambulance, %	1,502 (27.8)	1,030 (27.3)	472 (29.1)	0.161
Unconscious, %	28 (0.5)	19 (0.5)	9 (0.6)	0.806
Respiratory rate	18 (16, 20)	18 (16, 20)	18 (16, 20)	0.278
Heart rate, bpm	75 (64, 87)	74 (64, 86)	75 (65, 88)	0.080
SBP, mmHg	139 (121, 155)	138 (121, 155)	139 (122, 155)	0.915
DBP, mmHg	81 (71, 92)	81 (71, 92)	71 (82, 91)	0.660
Hypotension (SBP <100 mm Hg), %	265 (4.9)	188 (5.0)	77 (4.8)	0.727
Killip class	/	/	/	0.644
I	4,982 (92.3)	3,496 (92.6)	1,486 (91.7)	/
II	261 (4.8)	180 (4.8)	81 (5.0)	/
III	74 (1.4)	48 (1.3)	26 (1.6)	/
IV	80 (1.5)	53 (1.4)	27 (1.7)	/
ECG ischaemia, %	2,587 (47.9)	99 (6.7)	2,488 (63.4)	0.421
Time from symptom onset to arrival in the ED, min	204 (92, 512)	210 (95, 529)	192 (87, 485)	0.020*
TAT of cTnI, min	16 (15,19)	16 (15, 19)	16 (15, 19)	0.828
POC cTnI, ng/ml	0.11 (0.05, 1.73)	0.11 (0.05, 1.64)	0.10 (0.05, 1.89)	<0.001*
hs-cTnI, ng/ml	9.34 (1.57, 29.30)	8.71 (1.46, 28.86)	10.53 (1.91, 31.13)	0.070
AMI, %	3,926 (72.7)	2,737 (72.5)	1,189 (73.4)	0.482
GRACE score	111 (91, 131)	111 (91,131)	112 (91,132)	0.632
30-day death	81 (1.50)	58 (1.54)	23 (1.42)	0.808

ED, emergency department; TAT, turnaround time; POC, point-of-care; IQR, inter-quartile range; SBP, systolic blood pressure; DBP, diastolic blood pressure; GRACE, global registry of acute coronary events.

* *p* < 0.05.

**Figure 2:**
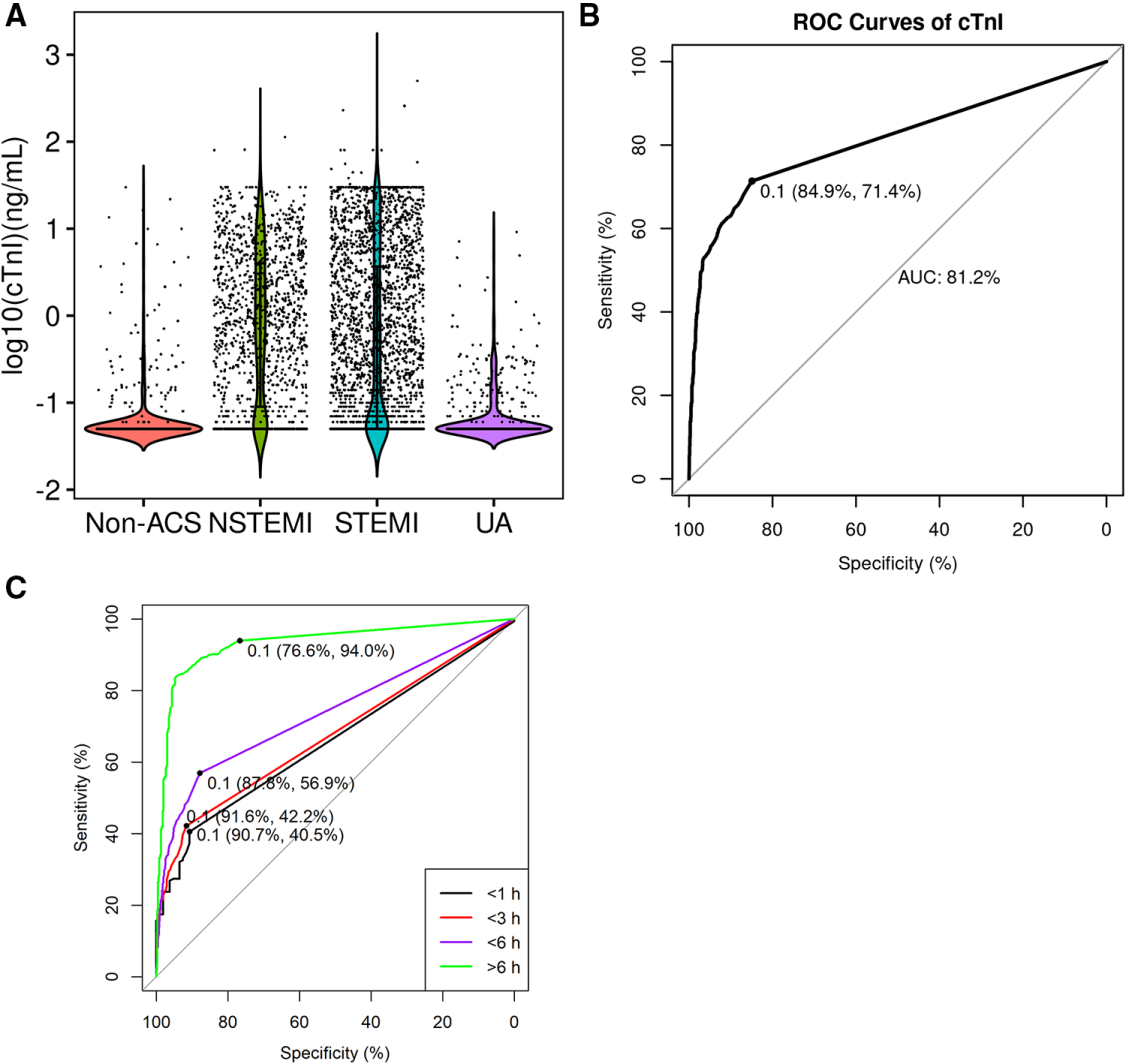
ROC curve of cTnI for AMI diagnosis. (**A**) Violin plot of point-of-care cTnI concentration levels in different groups of patients; (**B**) ROC curve of cTnI for AMI diagnosis in all patients; (**C**) ROC curve of cTnI for AMI diagnosis in different time points. STEMI, ST-segment elevation myocardial infarction; NSTEMI, non-ST-elevation myocardial infarction; UA, unstable angina pectoris; AMI, acute myocardial infarction.

The cTnI levels of each group are shown using a violin plot in Figure 2A, and the AUC of cTnI for AMI diagnosis was 81.2% (Figure 2B). POC cTnI alone showed inadequate sensitivity for AMI diagnosis (sensitivity: 71.4% and specificity: 84.9%), especially in the early onset of chest pain (Figure 2C). The clinicians could make an initial diagnosis of AMI or not for timely decision and management of the patients, and the diagnosis may be made even before the cTnI assay. We found that 95.3% of the AMI were correctly diagnosed, while 13.6% of the non-AMI patients were misdiagnosed with AMI. The management of ACS included 4 categories: (a) No invasive manipulation, (b) CAG only without cardiac surgery, (c) CAG with emergency cardiac surgery, including percutaneous coronary intervention (PCI) or coronary artery bypass grafting (CABG), and (d) CAG with undergoing elective cardiac surgery. Most of the AMI patients (50.7%) had undergone elective surgery. According to research, troponin has a window period, suggesting that construction of prediction model may fill the gap when combined troponin with ECG and other variables.

**Figure 3:**
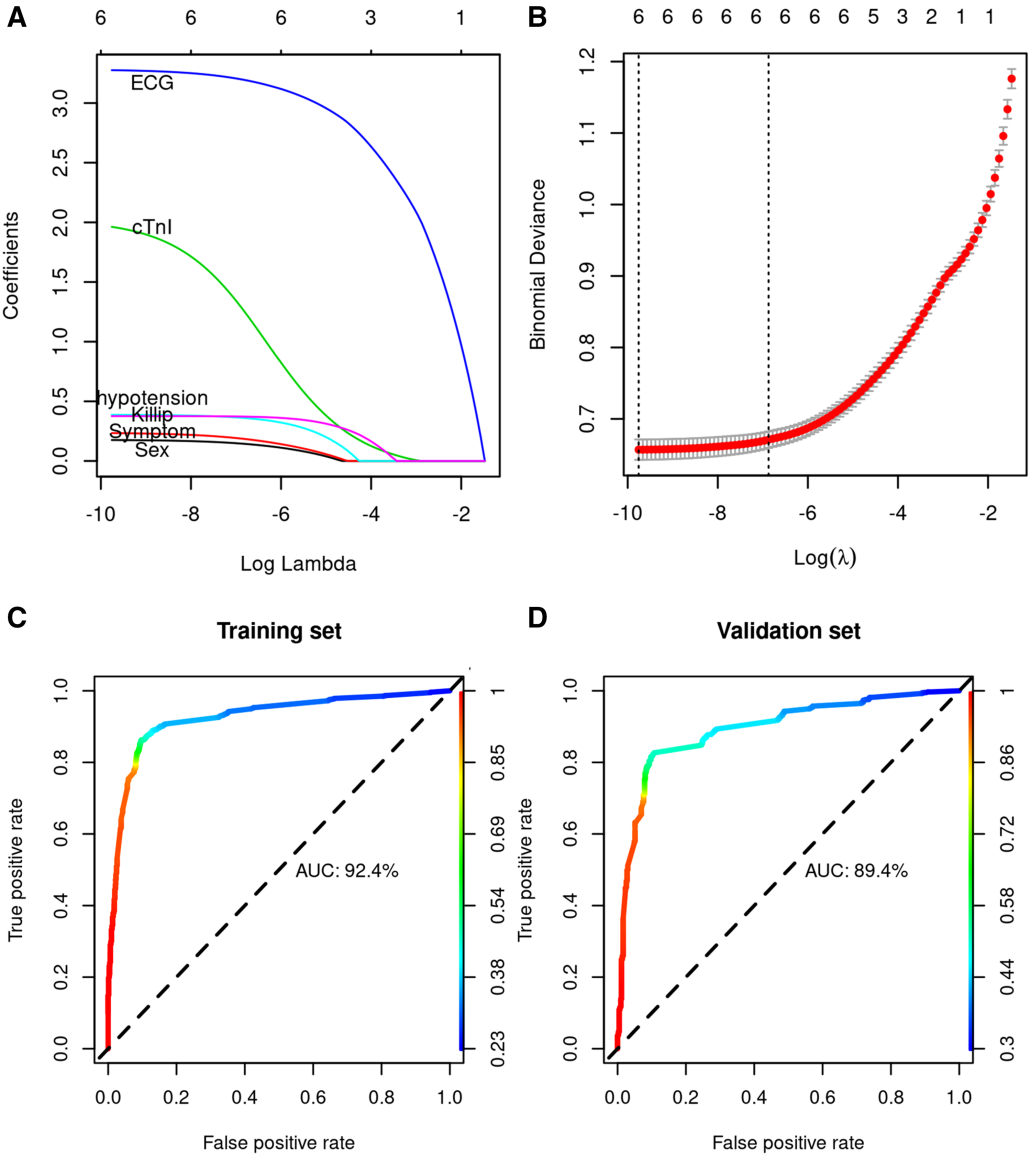
LASSO model profile plots. (**A**) LASSO coefficient profiles of the 6 variables showing how the size of the coefficients associated with increasing value of the lambda penalty; (**B**) penalty plot for the LASSO model; color error bars indicate the standard error; (**C**) ROC curve of the prediction model for AMI diagnosis in the training cohort; (**D**) ROC curve of the prediction model for AMI diagnosis in the validation cohort.

Logistic regression analysis identified factors significantly associated with AMI, including ECG ischemia, cTnI, chest pain symptoms, Killip class, hypotension, and sex (Table 2). Then we used the LASSO regression model to build a diagnostic classifier that included the six variables above (Figure 3). In the training set, the AUC was 92.4%, and in the validation set, the AUC was 0.894 (Figure 3), indicating it can have a good diagnostic performance for acute chest pain triage.

**Table 2:** Management of all patients.

Characteristic	Total (*n* = 5,397)	Non-AMI (*n* = 1,471)	AMI (*n* = 3,926)	*p* value
Initial diagnosed with AMI	3,942 (73.0)	200 (13.6)	3,742 (95.3)	<0.001*
Treatment, %	/	/	/	<0.001*
No invasive manipulation	2,894 (53.6)	1,348 (91.6)	1,546 (39.4)	/
CAG only	261 (4.8)	56 (3.8)	205 (5.2)	/
CAG + Emergency surgery^a^	184 (3.4)	5 (0.3)	179 (4.6)	/
CAG + Elective surgery^a^	2,058 (38.1)	62 (4.2)	1,996 (50.7)	/
30-day death	81 (1.50)	58 (15.4)	23 (14.2)	0.808

CAG, coronary arteriography; PCI, percutaneous coronary intervention; CABG, coronary artery bypass grafting.

^a^ The emergency or elective surgery refers to PCI or CABG.

* *p* < 0.05.

**Figure 4:**
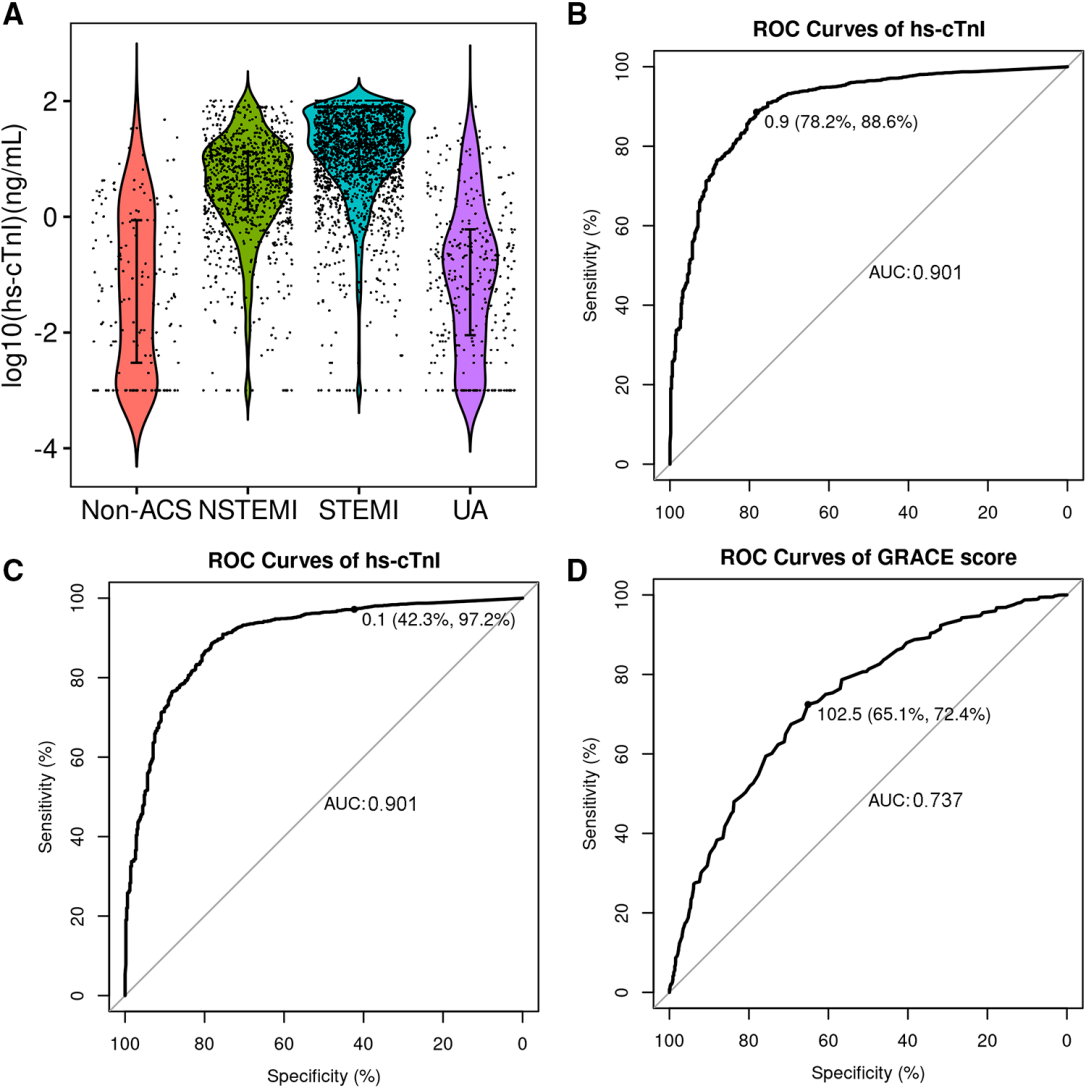
The diagnostic performance of high sensitivity (hs)-cTnI and GRACE score. (**A**) Violin plot of hs-cTnI concentration levels in different groups of patients; (**B**) ROC curve of hs-cTnI for AMI diagnosis in all patients with the cut-off value determined by Youden index; (**C**) ROC curve of hs-cTnI for AMI diagnosis in all patients with the cut-off value same as POC cTnI; (**D**) ROC curve of the GRACE score for AMI diagnosis in all patients.

To investigate if the nomogram adds value over and beyond existing clinical strategies for risk stratification of chest pain, we compared it with hs-cTnI and the Global Registry of Acute Coronary Events (GRACE) score. The hs-cTnI level is shown in Figure 4A. The hs-cTnI has a similar performance (AUC 0.901, Figure 4B) to the model with better sensitivity to POC cTnI. However, the time of collecting blood samples of hs-cTnI was later than POC cTnI as the hs-cTnI was much higher than POC cTnI (Table 1). When using the same cut-off (0.1 ng/ml) with POC cTnI, the specificity is only 42.3% (Figure 4C). The major advantage of hs-cTnI is a smaller LoD. The LoD of POC cTnI is 0.05 ng/ml, which is lower than the cut-off value. Using a lower cut-off would result in a higher false positive rate. The GRACE score has a relatively low AUC (0.737, Figure 4D), indicating the model has better prediction performance than the GRACE score.

## Discussion

In the study, we constructed a clinical prediction model for rapid AMI diagnosis using the data from a population of 5,397 chest pain patients. The early AMI diagnosis has an important clinical significance which may reduce the rate of mortality and comorbidity and reduce unnecessary medical costs. The T-MACS decision rule is a common clinical prediction model for ACS (12, 13, 26, 27) which consisted of hs-cTnT and heart-type fatty acid binding protein (H-FABP), ECG, and clinical symptoms, but H-FABP was not routinely evaluated in CPCs. The classification of symptoms in CPC is also different from the T-MACS model. Typical chest pain of AMI may be accompanied by sweating and pain radiating to the left arm or shoulder, and some patients have nontypical symptoms such as stomachache or vomiting. The status of chest pain (nontypical, relieved, persistent, or intermittent) is routinely assessed and recorded in the CPC, so we constructed a model for the CPC which is adapted from the T-MACS model. There are also other modes such as the clinical chemistry score (CCS) (28). The ESC 0 h/1 h algorithm based on hs-cTnI has been established; however, the algorithm is assay-specific (29) and not suitable for POC assays. POC cTnI assay is routinely used in CPC as the median TAT is 16 min (Table 1), and it is faster than hs-cTnI although serial hs-cTnI/T assay was also performed in the clinical laboratory. A meta-analysis showed the POC assay could make clinical decisions 40 min faster (30). Currently, available models may not be suitable for the population in CPCs of China, and, therefore, we need to build our custom prediction model.

We have identified several variables other than troponins, such as ECG and hypotension. ECG has an important role in AMI diagnosis, especially sensitivity in STEMI, which is an important supplement for troponin. We found that men have a higher AMI risk (Table 3), which is a known factor of obstructive coronary artery disease (31). POC cTnI alone is not sensitive to AMI with a sensitivity of 71.4%. Recently, Stopyra (32) reported POC cTn has a higher specificity but low sensitivity, which was similar to our results. Still, several studies reported POC cTn alone to have good diagnostic performance for suspected cardiac chest pain (33, 34). Using the six variables, we constructed a model with better sensitivity and specificity rather than using POC cTnI alone (Figures 2B,C, 3). The high sensitivity enables rapid rule out of AMI patients, and the AMI patients ruled in may have an earlier invasive approach, which reduces care time in ED.

**Table 3:** Multivariate logistic regression model of the training cohort.

Variable	*B*	OR (95% CI)	*p* value
a. Sex	0.1787	1.196 (0.996, 0.148)	0.100
Symptom	/	/	/
b. Intermittent chest pain	0.2651	1.304 (1.012, 1.681)	0.041
c. Persist chest pain	0.4811	1.618 (1.233, 2.124)	<0.001
d. cTnI	2.0425	7.710 (5.701, 10.730)	<0.001
e. ECG	3.2827	26.649 (20.559, 35.032)	<0.001
f. Hypotension	0.5430	1.721 (0.985, 3.083)	0.061
g. Killip class	0.3676	1.444 (1.136, 1.860)	0.003
(Intercept constant)	−1.6881	/	/

The model estimates the probability (*p*) of AMI as follows: *p* = 1/[1 + exp(0.068a + 0.2651b + 0. 4811c + 2.0425d + 3.2827e + 0.5430f + 0.3676g − 1.6881)]. For each categorical variable, a value of 1 is assigned if the characteristic is present.

The early diagnosis leads to an early clinical decision, most (95.3%) of AMI patients could be diagnosed at the initial time (Table 3). Early AMI diagnosis is important as it could improve the outcome of AMI (35–37) and reduce medical costs of non-AMI, and the clinical prediction model may enable fast diagnosis. However, we wonder whether early invasive therapy on AMI patients has been made. Research has found that there is a treatment paradox in ACS because ACS patients with high mortality risk may have worse status, such as advanced age, renal failure, or heart failure. The clinicians preferred not to perform invasive manipulation in these high-risk patients; however, these patients are more likely to benefit from invasive therapy (38, 39). Our result showed 4.6% of the AMI patients and 0.3% of the non-AMI patients had undergone emergency surgery. However, about half of the AMI patients had undergone elective cardiac surgery; we think this may be similar to the previously reported treatment paradox.

The sensitivity of the POC cTnI assay is low. To improve the sensitivity without increasing the TAT, we think a more sensitive POC cTn assay is warranted in the CPC. In this study, a triage product was used, which was a common assay used in many studies (40, 41). A high-sensitive cTnI has a better sensitivity which could rule out AMI for 0/1 h and 3 h evaluation (42) according to the European guidelines. There are also studies that compare different POC or high-sensitive assays (34, 43–45). Most POC cTnI assays had an excellent correlation with central laboratory assays. This indicates POC cTn assays could also be sensitive for AMI in the early stage. Durie (46) evaluated the performance of five POC cTn and found that not all POC assays were sensitive.

The prevalence of AMI in this study was 72.7%, which is much higher than in similar studies. Aldous (34) reported about 23% of 962 chest pain patients in ED had AMI. Shah (47) reported 21.4% of 48,282 suspected ACS patients had a myocardial injury. This is because prehospital triage was performed. The inclusion and exclusion criteria are not unbiased, leading to the unusually high prevalence of AMI in this study. The PPV and NPV values were affected by the prevalence of AMI, therefore, causing a relatively higher PPV and lower NPV compared with the previous report (12). Therefore, the model does not fit a general emergency department. However, this is a real-world study. We think the condition (prehospital triage) is also common in many other CPCs; hence, this model could be practical for other CPCs.

The study has several limitations. This is a retrospective study, and we analyzed several known risk factors of AMI, which are routinely documented in the CPC. We did not compare with some other known models such as the TIMI and heart scores as we could not get complete data for these analyses.

## Conclusion

A practical predictive model was created and could be used for rapid and effective triage of acute chest pain patients in the CPC.

## Data Availability

The raw data supporting the conclusions of this article will be made available by the authors, without undue reservation.
